# Milk-Derived Extracellular Vesicles Inhibit *Staphylococcus aureus* Growth and Biofilm Formation

**DOI:** 10.3390/ani16010123

**Published:** 2026-01-01

**Authors:** Peng Liu, Zhaoyuan Wang, Ziqiang Gao, Juan Liu, Yutong Zhang, Yangyang Song, Xiaolin Li, Huaxue Song, Xingli He, Fanzhi Kong, Changyuan Wang, Binglei Shen

**Affiliations:** 1 College of Animal Science and Veterinary Medicine, Heilongjiang Bayi Agricultural University, Daqing 163319, China; 18346663533@163.com (P.L.); zhaoyuan5260@163.com (Z.W.); 13333287067@163.com (Z.G.); 13136755125@163.com (Y.Z.); 15846397618@163.com (Y.S.); lxl13795131329@163.com (X.L.); 13359929549@163.com (H.S.); hxl98747500@163.com (X.H.); fanzhikong110@hotmail.com (F.K.); 2 Yi’an County Vocational and Technical Education Center School, Qiqihaer 161500, China; liuxinan960401@163.com; 3 College of Food Science, Heilongjiang Bayi Agricultural University, Daqing 163319, China; byndwcy@163.com

**Keywords:** bovine mastitis, milk-derived extracellular vesicles, *Staphylococcus aureus*, biofilm formation, miRNA regulation, antimicrobial activity, Host—pathogen interactions

## Abstract

*Staphylococcus aureus* is a key pathogen in bovine mastitis, and antibiotic therapy is challenged by resistance and residue concerns. Milk-derived extracellular vesicles emerge as promising natural antimicrobials. This study aimed to evaluate the antimicrobial activity and explore potential associated mechanisms of milk-derived extracellular vesicles against *S. aureus*. Milk-derived EV-enriched fractions (mEVs) from healthy (HmEVs) and mastitic (MmEVs) bovine milk suppressed *S. aureus* growth in vitro and were associated with oxidative imbalance, with MmEVs showing stronger inhibition. In addition, MmEVs significantly reduced biofilm biomass, extracellular matrix production, and the expression of key biofilm-associated genes (*sarA*, *icaB*, *fnbA*, *clfB*, *cidA*). Small RNA sequencing revealed distinct miRNA profiles between HmEVs and MmEVs; in particular, MmEVs were enriched in miRNAs predicted to target the *S. aureus* biofilm-associated gene *clfB*. Although we did not directly demonstrate uptake of mEV-derived miRNAs by bacteria or their regulation of bacterial gene expression in this study, our small RNA sequencing data together with subsequent bioinformatic predictions suggest that vesicular miRNAs should be regarded as candidate contributors, rather than demonstrated mediators, of the observed antibacterial and antibiofilm effects. Taken together, these findings indicate the potential of mEVs as residue-free adjuncts for controlling bovine mastitis, while recognizing that the present conclusions are mainly derived from in vitro experiments with *S. aureus* and bioinformatic analyses. Therefore, functional validation of candidate miRNAs, in vivo studies, and evaluation of activity against other mastitis-associated pathogens are still required to clarify the underlying mechanisms, therapeutic potential, and spectrum of activity of mEVs.

## Simple Summary

Bovine mastitis is a common and costly disease in dairy cows, caused by *Staphylococcus aureus*, which is hard to treat due to its ability to form biofilms and resist antibiotics. In this study, we explored the potential of milk-derived extracellular vesicles as natural antimicrobial agents. We compared exosomes from healthy and mastitic milk and found that both inhibited bacterial growth and were associated with disrupted oxidative balance, with stronger effects from mastitic milk. These vesicles also reduced biofilm formation and were associated with lower expression of biofilm-related genes. Small RNA sequencing suggested that vesicular miRNAs might be involved in these effects, and mastitic milk-derived vesicles were enriched in miRNAs predicted to target biofilm-associated genes; however, bacterial uptake and direct functional regulation of these miRNAs were not assessed in this study. Our findings indicate that milk-derived extracellular vesicles have potential as residue-free adjuncts for controlling bovine mastitis, but these conclusions are based on in vitro data and require further validation in animal models before clinical application.

## 1. Introduction

*Staphylococcus aureus* (*S. aureus*) is a major opportunistic pathogen associated with a wide range of infectious diseases in both humans and animals, including bovine mastitis [1]. This bacterium employs multiple strategies, such as biofilm formation and immune evasion, to withstand host defenses [2,3,4]. Current therapeutic approaches rely heavily on antibiotics [5]; however, their efficacy is increasingly compromised by the global rise in antimicrobial resistance (AMR) [6]. In addition, antibiotic residues in milk and dairy products raise concerns regarding consumer health and undermine milk safety standards [7,8]. Consequently, there is an urgent need for innovative, effective, and residue-free alternatives to control *S. aureus* infections.

Extracellular vesicles (EVs) are nanoscale, membrane-bound particles secreted by nearly all cell types, carrying diverse bioactive cargos such as proteins, lipids, and RNAs including mRNAs, lncRNAs, and microRNAs (miRNAs) [9,10]. By transferring these cargos to recipient cells, EVs serve as important mediators of intercellular communication and regulators of physiological and pathological processes [11]. Recent evidence indicates that EVs also participate in host–pathogen interactions [12,13]. Studies have shown that natural extracellular vesicles (exosomes) derived from various biological sources, including bee products, foods, human urine and bovine milk, possess antimicrobial activity such as inhibition of bacterial growth and suppression of biofilm formation. These vesicles can inhibit multiple Gram-positive and Gram-negative bacteria, such as *Escherichia coli* and oral streptococci, and can even attenuate adherent-invasive *E. coli* infection in vivo. Together, these findings highlight the broad-spectrum antimicrobial potential of EVs and their role as innate antimicrobial mediators [14,15,16,17], although the molecular mechanisms underlying these effects remain insufficiently characterized.

Milk is an especially rich source of EVs, with concentrations reaching up to 10^12^ particles per milliliter [18]. While milk-derived EVs have been investigated in nutritional and immunological contexts [19], their role in bacterial pathogenesis and infectious disease regulation remains poorly defined. Emerging evidence indicates that the biogenesis and cargo composition of extracellular vesicles are influenced by the physiological or pathological status of the donor cells. EVs released under inflammatory or infectious conditions often display distinct RNA and protein profiles and exert altered immunomodulatory or antimicrobial activities compared with EVs from healthy controls [20,21]. These observations motivated us to investigate whether disease-associated changes in mEV cargo influence their interaction with *S. aureus*.

Building on these observations, this study explores the antimicrobial potential of mEVs against *S. aureus* by systematically assessing their effects on bacterial growth, oxidative stress, biofilm development, and gene expression. Furthermore, by comparing milk-derived EV-enriched fractions (mEVs) isolated from healthy and mastitic milk (HmEVs and MmEVs) and profiling their miRNA cargo using small RNA sequencing, we aim to identify candidate functional components that may contribute to these antibacterial effects. These findings are expected to advance our understanding of mEVs as natural antimicrobial nanovesicles and provide initial insights into their role in host–pathogen interactions, thereby supporting the exploration of residue-free strategies for infection control and contributing to efforts against antimicrobial resistance.

## 2. Materials and Methods

### 2.1. Experimental Animals and Sample Collection

Holstein cows were selected as experimental subjects from a dairy farm in Daqing City, Heilongjiang Province. Based on data from the Dairy Herd Improvement (DHI) program, a total of 6 healthy cows and 6 cows with mastitis were selected for the study. Cows were assigned to the mastitis group if they had a recorded episode of clinical mastitis in the current lactation and presented typical clinical signs at sampling, including udder inflammation (swelling, heat or pain of the affected quarter) and abnormal milk appearance (flakes, clots or watery secretion), together with elevated somatic cell counts (SCC > 5 × 10^5^ cells/mL) in the DHI records. Cows in the healthy group had no history or clinical signs of mastitis during the current lactation and exhibited SCC values within the normal range (SCC < 2 × 10^5^ cells/mL) in at least the two most recent DHI tests; cows that did not meet these criteria were excluded from the study. Milk samples from all selected cows were aseptically collected for further experimental analysis and were immediately cooled on ice. For cows with clinical mastitis, each milk sample was divided into two portions: one for EVs isolation and the other for the isolation of clinical *S. aureus* strains. Milk samples from healthy cows were used exclusively for EVs isolation and were not subdivided.

### 2.2. Extracellular Vesicles Extraction and Characterization

Milk-derived EV-enriched fractions (mEVs) were isolated from raw milk as described by Luo et al. (2023) with minor modifications [22]. Briefly, fresh milk was centrifuged at 3000× *g* for 30 min at 4 °C to remove somatic cells, fat globules, and other debris. The supernatant was treated with chymosin to induce casein coagulation, and the coagulated casein and precipitates were removed by centrifugation at 10,000× *g* for 30 min. The clarified supernatant was then ultracentrifuged at 170,000× *g* for 90 min to pellet small EVs. Pellets were resuspended in PBS, passed through 0.45 μm and 0.22 μm syringe filters (Sartorius, Göttingen, Germany), and centrifuged again at 170,000× *g* for 90 min to remove residual proteins and large particles. The final EV-containing pellets were resuspended in PBS and used as mEVs for subsequent experiments and stored at −80 °C until further analysis. Morphology was examined by transmission electron microscopy (TEM) with uranyl acetate negative staining. Particle size distribution was determined by Nano Flow Cytometry (NanoFCM). Exosomal marker proteins CD9 and TSG101 (Abclonal, Wuhan, China) were detected by Western blot. This differential centrifugation and filtration protocol yields EV-enriched fractions that substantially deplete casein micelles and cellular debris, as reported for similar milk EV preparations, and is intended to provide EV-enriched rather than fully purified vesicle preparations.

### 2.3. Isolation and Identification of Clinical Strains of S. aureus

Milk samples were centrifuged (4000× *g*, 10 min, 4 °C), and the precipitates were resuspended in saline and plated on blood agar. After 24 h incubation at 37 °C, single colonies were purified. Isolates were identified by Gram staining and by PCR amplification of the *Nuc* and *16S rDNA* genes, and the PCR products were confirmed using agarose gel electrophoresis. The *nuc* gene encodes a thermostable nuclease and is widely used as a species-specific molecular marker for the genotypic identification of *S. aureus*. The primer sequences were the following: *Nuc* forward 5′-GCGATTGATGG TGATACGGTT-3′, *Nuc* reverse 5′-AGCCAAGCCTTGACGAACTAAAGC-3′, *16S rDNA* forward 5′-AGAGTTTGATCMTGGCTCAG-3′, *16S rDNA* reverse 5′-AAGGAGGTGAT CCAGCCGCA-3′.

### 2.4. Bacterial Culture and mEV–S. aureus Interaction Assay

The standard strain *S. aureus* BNCC186335 (Beina Chuanglian, Beijing, China) was stored in glycerol at −80 °C. This strain was used as a reference in the bacterial growth inhibition and ROS assays to enable comparison with mastitis-associated clinical isolates. Biofilm inhibition assays were performed using clinical isolates only. Both standard and clinical *S. aureus* strains were cultured in LB broth at 37 °C. mEVs were labeled with PKH26 fluorescent dye (Solarbio, Beijing, China) according to the manufacturer’s protocol. The labeled mEVs were then subjected to ultracentrifugation (120,000× *g* for 90 min), resuspended, and co-cultured with *S. aureus* at a final concentration of 500 μg/mL for 6 h. For the dye-only control (NC + PKH26), *S. aureus* was incubated with PKH26 in PBS under the same conditions without mEVs and processed in parallel to control for artifacts caused by free dye or non-vesicular components. Afterward, the cells were counterstained with DAPI (Beyotime, Shanghai, China), washed, and observed under a fluorescence microscope (IX70, Olympus, Tokyo, Japan).

### 2.5. Determination of Effective mEVs Concentrations and Growth Curve Analysis

To determine the minimum inhibitory concentration (MIC) of mEVs against *S. aureus*, overnight cultures of the standard strain were diluted in LB broth to a standard inoculum and incubated with HmEVs or MmEVs at final concentrations of 0, 62.5, 125, 250, 500, 1000, and 2000 μg/mL at 37 °C with shaking for 24 h. After incubation, the turbidity and bacterial pellet of each culture were recorded, and bacterial growth was measured at 620 nm (OD_620_). The MIC of each mEV preparation against the standard *S. aureus* strain was defined as the lowest mEV concentration at which bacterial growth was clearly inhibited compared with the untreated control, based on both visual inspection and OD_620_ readings.

The inhibitory effects of mEVs from healthy (HmEVs) and mastitic (MmEVs) cows on *S. aureus* were evaluated by gradient dilution. Bacterial suspensions were treated with mEVs at 2 × MIC, MIC, and 1/2 MIC, and incubated at 37 °C with shaking for 24 h. Growth inhibition was assessed by measuring OD_620_.

### 2.6. Reactive Oxygen Species (ROS) Detection

*S. aureus* cells in logarithmic phase were harvested (5000× *g*, 10 min, 4 °C), resuspended, and treated with HmEVs and MmEVs (250 or 500 μg/mL) for 6 h at 37 °C. Cells were then incubated with 10 μM DCFH-DA for 20 min in the dark, washed, and adjusted to 1 × 10^8^ CFU/mL. ROS fluorescence was visualized by microscopy and quantified using ImageJ 1.54c.

### 2.7. Enzyme Activity Assays

SOD, CAT, POD, and ATPase activities in *S. aureus* were measured using commercial assay kits (Solarbio, Beijing, China) following the manufacturer’s instructions.

### 2.8. Biofilm Formation Assay

To assess biofilm inhibition, *S. aureus* suspensions (1.0 × 10^5^ CFU/mL) were incubated with MmEVs (62.5–2000 μg/mL) in 96-well plates at 37 °C for 18 h. Biofilm biomass was quantified by crystal violet staining, and inhibition rates were calculated as:

Inhibition (%) = [(OD570 control − OD570 treated)/OD570 control] × 100.

### 2.9. Scanning Electron Microscopy (SEM)

*S. aureus* cells were incubated with or without MmEVs at the MBIC for 8 h at 37 °C, prepared for SEM by standard procedures, and imaged under a scanning electron microscope.

### 2.10. Physicochemical Tolerance Assays

Acid tolerance was tested by culturing *S. aureus* in TSB (pH 5.0) with or without MmEVs for 4 h at 37 °C, followed by serial dilution, TSA plating, and CFU counting.

Heat tolerance was assessed by exposing MmEV-treated cultures to 58 °C for 5 min before dilution and plating.

Cell surface hydrophobicity was measured by microbial adhesion to hydrocarbons (MATH) assay using n-hexadecane, with hydrophobicity calculated as [(ODA − ODB)/ODA] × 100.

Electrolyte leakage was evaluated by monitoring culture conductivity at 0–8 h at 37 °C using a conductivity meter.

### 2.11. Biofilm Extracellular Components

Mature biofilms were cultured for 24 h, washed with PBS, and treated with PBS, proteinase K (100 μg/mL), sodium periodate (10 mM), or DNase I (24 μg/mL) for 2 h at 37 °C. Biofilm biomass was measured by crystal violet staining (OD570). eDNA was extracted from biofilms treated with PBS, MBIC- or 1/2 MBIC-dose MmEVs using a commercial kit (Solarbio, China) and quantified spectrophotometrically (A260). Extracellular proteins were measured by BCA assay (OD570), and polysaccharides by ethanol precipitation and phenol–sulfuric acid assay (OD490).

### 2.12. RT-qPCR Analysis

Total RNA was extracted from *S. aureus* using a commercial kit (Sangon Biotech, Shanghai, China) according to the manufacturer’s protocol. RT-qPCR was performed with TB Green Premix Ex Taq (Takara, Japan) on a CFX Connect Real-Time PCR system (Bio-Rad, Hercules, CA, USA). Gene expression levels were normalized to *16S rRNA* and calculated using the 2^−ΔΔCt^ method. The primer sequences were the following: *SarA* forward 5′-CAATTAGCTTTGAAGAATTCGCT-3′, *SarA* reverse 5′-TGCACCGTC-TTCTACCCAAG-3′, *icaB* forward 5′-ATACCGGCGACTGGGTTTAT-3′, *icaB* reverse 5′-TTGCAAATCGTGGGTATGTGT-3′, *FnbA* forward 5′-GATACAAACCC-AGGTGGTGG-3′, *FnbA* reverse 5′-TGTGCTTGACCATGCTCTTC-3′, *ClfB* forward 5′-GGTGGTGGAAGTGCTGATGGTG-3′, *ClfB* reverse 5′-CTTGGTTCTG-GATCTGGCGTTGG-3′, *CidA* forward 5′-ATCTTCCCTTAGCCGGCAGT-3′, *CidA* reverse 5′-TGCACCGTCTTCTACCCAAG-3′, *gyrB* forward 5′-GCCGATTGCTCTA-GTAAAAGTCC-3′, *gyrB* reverse 5′-GATTCCTGTACCAAATGCTGTG-3′, *16s-rRNA* forward 5′-GAAAGCCTGACGGAGCAA-3′, *16s-rRNA* reverse 5′-CGTTT-ACGGCGTGGACTA-3′.

### 2.13. Small RNA Sequencing

Total RNA, including small RNAs, was extracted from mEVs and subjected to high-throughput sequencing (LC-Bio Technology, Hangzhou, China). RNA quality control, library construction, and HiSeq/MiSeq sequencing were performed according to the manufacturer’s protocols.

### 2.14. Target Site Prediction

MiRNA seed sequences were aligned to the *S. aureus* genome using STarMir (https://sfold.wadsworth.org/cgi-bin/starmir.pl, (accessed on 27 September 2025). Potential targets with binding free energy (ΔG) < −15 kcal/mol were considered likely interactions.

### 2.15. Statistical Analysis

Data are presented as mean ± standard deviation (SD) from at least three independent experiments. Statistical analyses were performed using GraphPad Prism 8.0 (GraphPad Software, San Diego, CA, USA). For datasets involving three or more groups, group differences were evaluated using one-way analysis of variance (ANOVA) followed by Tukey’s multiple comparison test. *p* < 0.05 was considered statistically significant.

## 3. Results

### 3.1. Isolation and Characterization of mEVs

**Figure 1:**
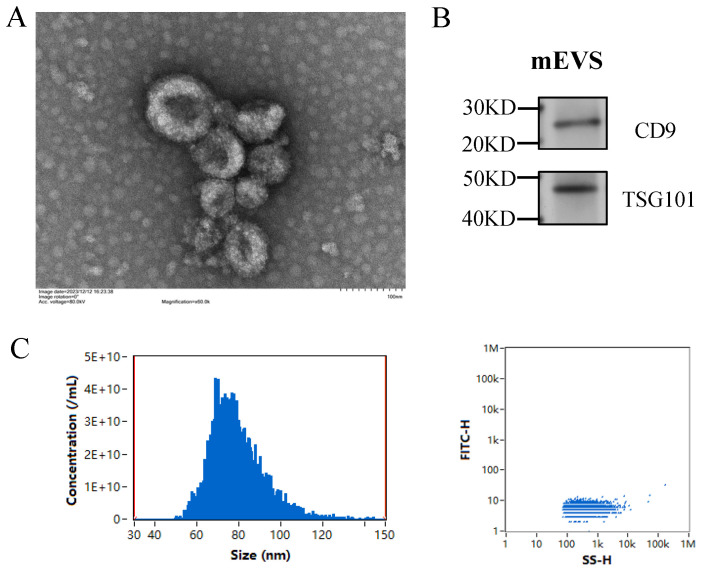
Identification of extracellular vesicles in bovine milks. (**A**) Representative TEM image of mEVs. (**B**) Western blotting analysis of CD9 and TSG101 in mEVs. (**C**) Particle size distribution of mEVs.

mEVs were isolated using a stepwise differential centrifugation and filtration procedure followed by ultracentrifugation and characterized by TEM, Western blotting, and NanoFCM. TEM revealed that mEVs exhibited the typical cup-shaped morphology of exosomes with diameters of 50–100 nm (Figure 1A). Western blotting confirmed the presence of exosomal markers CD9 and TSG101 (Figure 1B). NanoFCM analysis revealed that the mean particle sizes of HmEVs and MmEVs were nearly identical, at 79.2 nm and 79.3 nm, respectively, indicating no obvious difference in particle size between the two mEVs populations (Figure 1C).

### 3.2. Isolation and Identification of Clinical Strains of S. aureus

**Figure 2:**
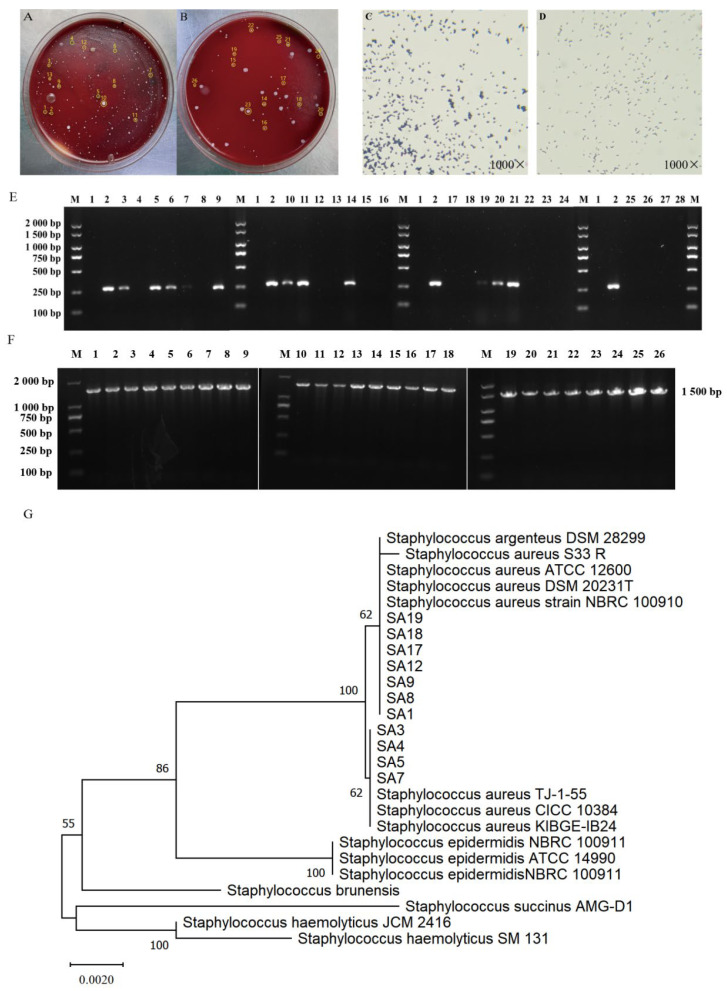
Identification of clinical strains of *S. aureus*. (**A**,**B**) Growth of pathogenic bacteria in milk samples of clinical and occult mastitis on a blood flat dish. (**C**,**D**) Results of Gram staining of clinical pathogenic bacteria in milk. (**E**) Identification of *Nuc* gene of clinical strains of *S. aureus*. (**F**) Identification of *16S rDNA* gene of clinical strains of *S. aureus*. (**G**) Evolutionary development tree of clinical and reference strains of *S. aureus*.

Milk samples were cultured on blood agar, and colonies showing typical *S. aureus* morphology (yellow/white, round, ~1–2 mm, with clear hemolytic rings) were selected (Figure 2A,B). Gram staining confirmed Gram-positive cocci (Figure 2C,D). PCR amplification of the *nuc* gene identified 11 isolates as *S. aureus* (Figure 2E). In addition, amplification of *16S rDNA* yielded ~1500 bp products in all 26 isolates (Figure 2F). Sequencing analysis showed that 11 isolates shared >99% sequence identity with reference *S. aureus* strains, while the remaining belonged to other *Staphylococcus species*. Phylogenetic analysis further confirmed that the 11 isolates clustered with reference *S. aureus* strains (Figure 2G). These clinical isolates were divided into two subgroups, SADQ1 and SADQ2, which were used as representative mastitis-associated *S. aureus* strains in the subsequent growth inhibition and ROS assays.

### 3.3. Interaction Between mEVs and S. aureus

**Figure 3:**
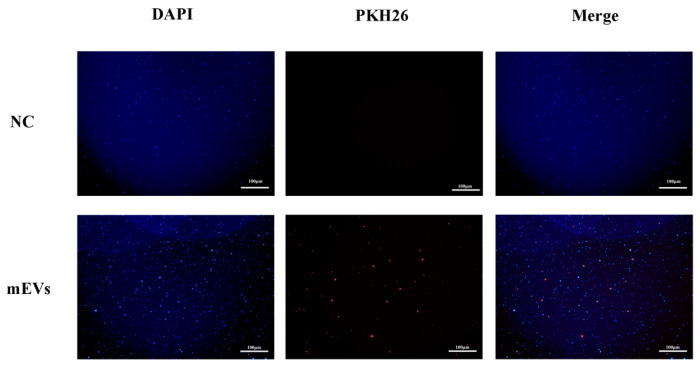
Representative fluorescence images of *S. aureus* incubated with PKH26 in the absence or presence of mEVs. NC, dye-only control (PBS + PKH26); mEVs, *S. aureus* incubated with PKH26-labeled mEVs.

PKH26-labeled mEVs were cocultured with *S. aureus*. In the mEVs + PKH26 group, fluorescence microscopy revealed distinct red signals closely associated with the bacterial cells, indicating a close association or interaction between mEVs and *S. aureus*. In contrast, no obvious red fluorescence was observed in the NC groups (PKH26-only control) (Figure 3).

### 3.4. Growth Inhibition of S. aureus by HmEVs and MmEVs

**Figure 4:**
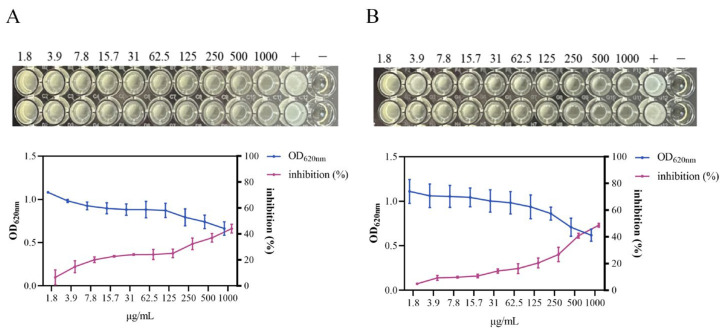
Dose–response curves of HmEVs and MmEVs on growth of the standard *S. aureus* strain. (**A**) Changes in OD_620_ and inhibition rate after 24 h treatment with increasing concentrations of HmEVs. (**B**) Corresponding dose–response curves for MmEVs under the same conditions.

In the dose–response assay, the standard *S. aureus* strain was incubated for 24 h with 0–2000 μg/mL of HmEVs or MmEVs. At concentrations of 500 μg/mL and above, both types of mEVs caused a clear reduction in the bacterial pellet and markedly decreased OD_620_ compared with the untreated control. Under these in vitro conditions, 500 μg/mL was therefore taken as the minimum inhibitory concentration (MIC) of HmEVs and MmEVs against the standard strain. Quantitatively, HmEVs at 500 and 1000 μg/mL reduced bacterial growth by 37.04% and 44.12%, respectively, whereas MmEVs at the same concentrations reduced growth by 40.86% and 48.72% (Figure 4), indicating that MmEVs exert slightly stronger antibacterial effects than HmEVs at equivalent protein concentrations.

**Figure 5:**
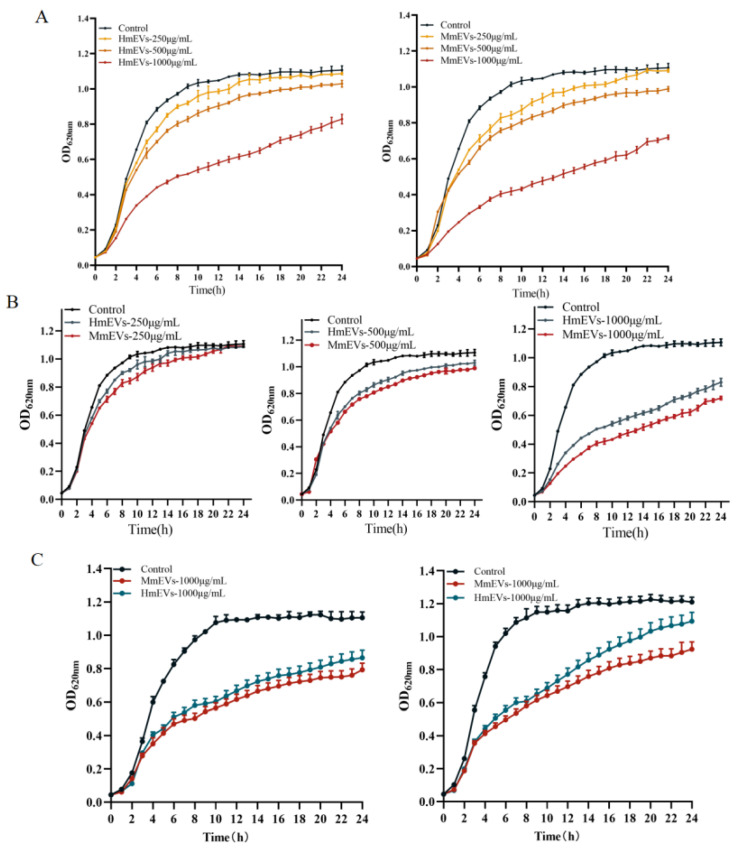
Growth curves of *S. aureus* treated with mEVs. (**A**) Standard strain exposed to increasing concentrations of mEVs. (**B**) Standard strain treated with HmEVs and MmEVs at the same concentration to compare their inhibitory effects. (**C**) Clinical isolates SADQ1 and SADQ2 treated with HmEVs and MmEVs at the same concentration.

Growth curve analysis showed that both HmEVs and MmEVs inhibited the proliferation of the *S. aureus* standard strain in a concentration-dependent manner, with the strongest inhibition observed at the highest mEV dose (Figure 5A). Figure 5B compares the effects of HmEVs and MmEVs on the standard strain at the same concentration and shows that MmEVs consistently exerted stronger suppression than HmEVs. Similar inhibitory patterns were observed in the clinical isolates SADQ1 and SADQ2 treated with HmEVs or MmEVs at the same concentration (Figure 5C).

### 3.5. Induction of ROS by HmEVs and MmEVs

**Figure 6:**
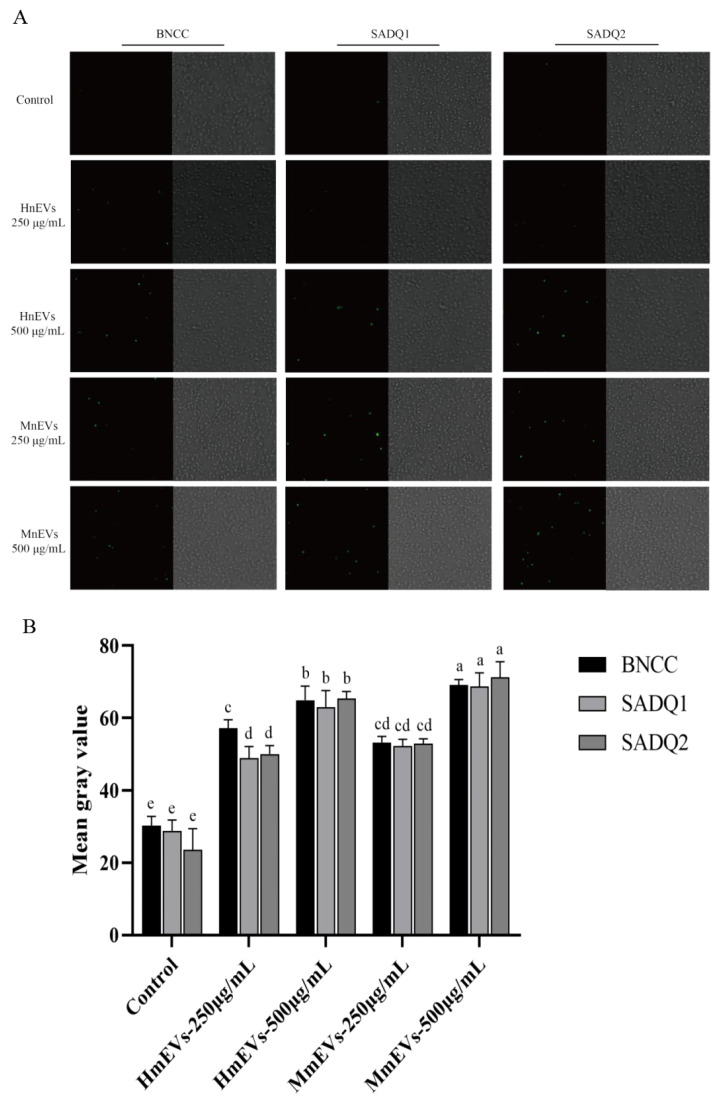
Intracellular ROS accumulation in *S. aureus* treated with mEVs. (**A**) Fluorescence microscopy images of ROS production in the standard strain, SADQ1, and SADQ2 treated with HmEVs or MmEVs. (**B**) Quantitative analysis of fluorescence intensity. For each *S. aureus* strain, bars representing different EV treatments that do not share a common lowercase letter differ significantly from each other (*p* < 0.05).

In the subsequent experiments assessing oxidative stress, we selected intermediate mEV concentrations of 250 and 500 μg/mL based on the growth-curve data. At 1000 μg/mL, mEVs produced near-maximal inhibition of bacterial growth and were therefore not used for ROS measurements. ROS quantification showed that untreated *S. aureus* exhibited baseline fluorescence of 27.6 ± 3.9 AU. Treatment with 250 μg/mL EVs increased ROS by 1.9-fold, while 500 μg/mL EVs induced a 5.1-fold increase. Notably, at 500 μg/mL, MmEVs produced 8.2% more ROS than HmEVs (Figure 6, *p* < 0.05).

### 3.6. Inhibition of Antioxidant and Metabolic Enzymes by HmEVs and MmEVs

**Figure 7:**
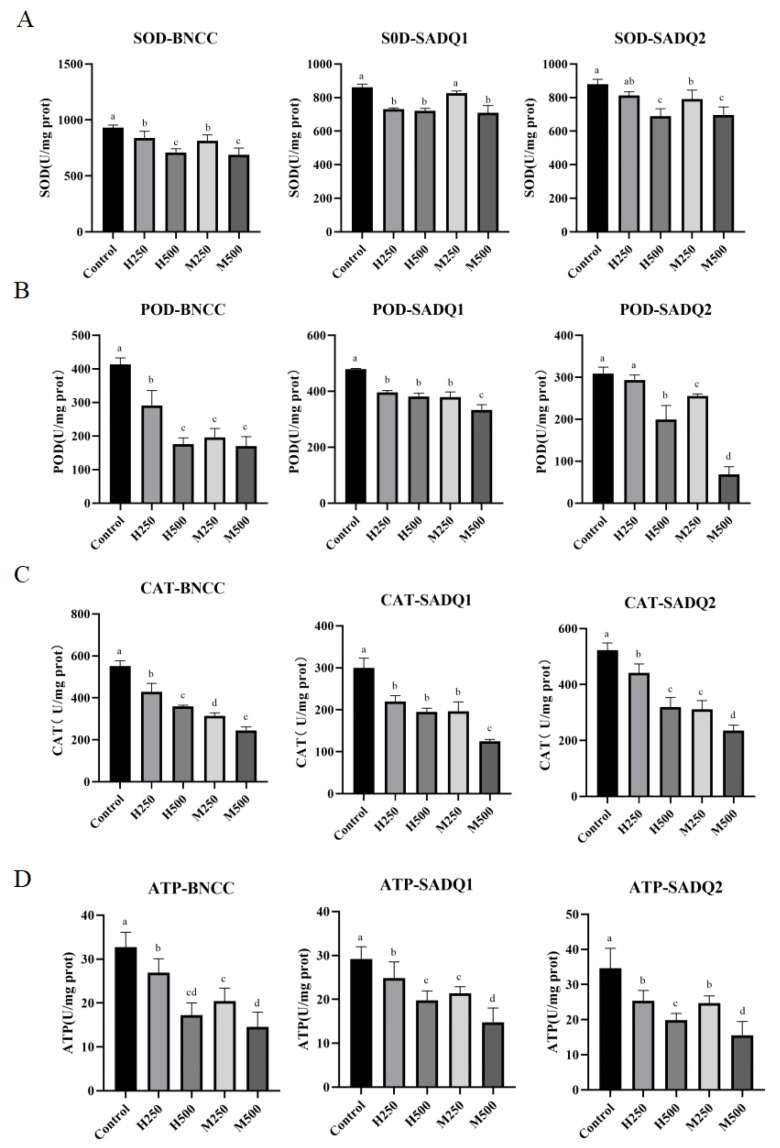
Effect of mEVs on SOD (**A**), CAT (**B**), POD (**C**) and ATPase (**D**) activity in *S. aureus*. Different lowercase letters indicate statistically significant differences among the groups within each panel (*p* < 0.05).

Enzyme activity assays demonstrated that both HmEVs and MmEVs inhibited SOD, CAT, POD, and ATPase activities in *S. aureus* in a dose-dependent manner, with higher concentrations leading to progressively stronger suppression (Figure 7, *p* < 0.05). MmEVs consistently exhibited greater inhibition compared with HmEVs. Since MmEVs showed stronger antimicrobial activity, subsequent experiments focused on their effects on biofilm formation and cell-envelope integrity.

### 3.7. Effects of MmEVs on S. aureus Biofilm and Ultrastructure

**Figure 8:**
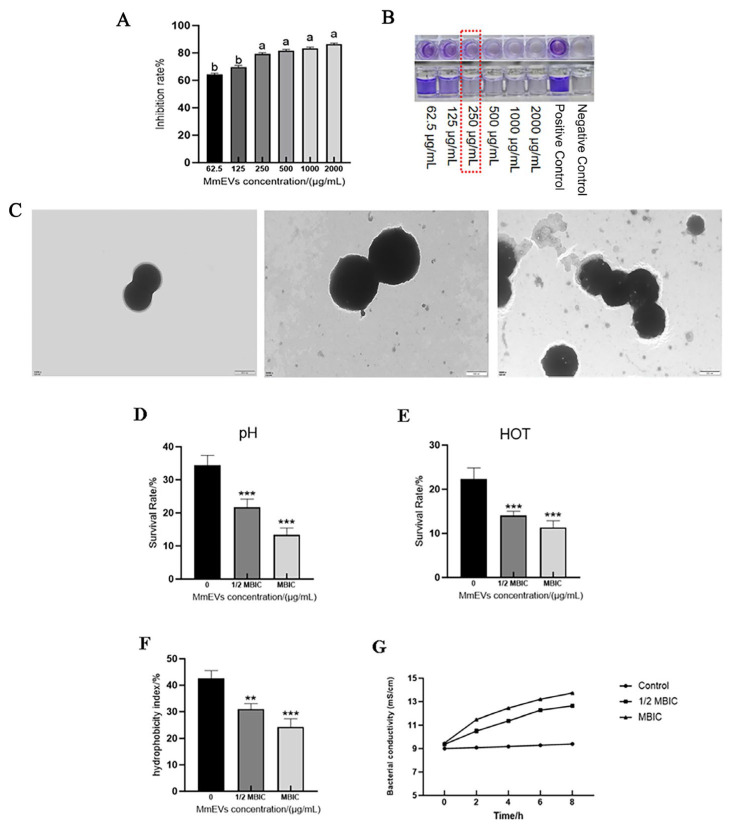
Effects of MmEVs on biofilm formation, ultrastructure, and physicochemical tolerance of *S. aureus*. (**A**) Biofilm biomass of *S. aureus* treated with different concentrations of MmEVs (62.5–2000 μg/mL), quantified by crystal violet staining. Different lowercase letters indicate statistically significant differences among the groups within each panel (*p* < 0.05). (**B**) Determination of the minimum biofilm inhibitory concentration (MBIC) of MmEVs. (**C**) Representative SEM images of *S. aureus* cells treated with MmEVs at the MBIC. (**D**) Survival of *S. aureus* under acidic conditions (pH 5.0) with or without MmEV treatment. (**E**) Survival of *S. aureus* after heat stress (58 °C, 5 min) with or without MmEV treatment. (**F**) Effect of MmEVs on cell surface hydrophobicity of *S. aureus* (**: *p* < 0.01, ***: *p* < 0.001). (**G**) Effect of MmEVs on the electrical conductivity of *S. aureus* suspensions over time.

Treatment with MmEVs (62.5–2000 μg/mL) suppressed biofilm formation in a concentration-dependent manner. Inhibition at 250 μg/mL was significantly greater than at 62.5 and 125 μg/mL (*p* < 0.05) but not different from higher concentrations (500–2000 μg/mL) (Figure 8A). Based on reduced crystal violet staining, the minimum biofilm inhibitory concentration (MBIC) was determined as 250 μg/mL (Figure 8B). SEM analysis revealed that *S. aureus* cells treated with MmEVs at the MBIC exhibited cytoplasmic leakage, indistinct envelope boundaries, and membrane detachment from the cell wall (Figure 8C). Crystal violet staining reflects total adherent biomass rather than specific matrix components, and the SEM images provide qualitative morphological evidence of MmEV-induced damage rather than direct proof of a particular molecular mechanism.

### 3.8. Effects of MmEVs on the Physicochemical Tolerance of S. aureus

MmEVs compromised the physicochemical tolerance of *S. aureus*. Under acidic conditions (pH 5.0, 4 h), survival rates in the 1/2 MBIC and MBIC groups were significantly lower than controls (Figure 8D, *p* < 0.001). Following heat stress (58 °C, 5 min), survival was also markedly reduced in both groups compared with the control (Figure 8E, *p* < 0.001). MmEVs significantly decreased cell surface hydrophobicity (Figure 8F, *p* < 0.01 and *p* < 0.001, respectively). Conductivity measurements showed time-dependent increases, with all MmEV concentrations significantly higher than control at each time point (Figure 8G). Together, these findings suggest that MmEVs impair stress tolerance and disrupt membrane integrity in *S. aureus*.

### 3.9. MmEV-Mediated Inhibition of Biofilm Extracellular Components and Gene Expression

**Figure 9:**
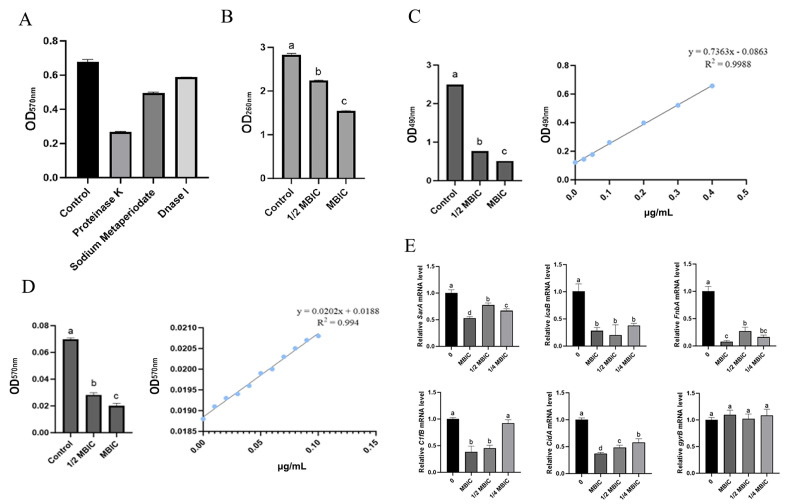
Effects of MmEVs on biofilm extracellular components and gene expression in *S. aureus*. (**A**) Enzymatic degradation of biofilm matrix components with proteinase K, sodium periodate, and DNase I. (**B**–**D**) Release of extracellular DNA (eDNA), proteins, and polysaccharides in *S. aureus* biofilms treated with different concentrations of MmEVs. (**E**) Expression levels of biofilm-associated genes (*sarA*, *icaB*, *fnbA*, *clfB*, *cidA*) and housekeeping gene (*gyrB*) measured by qRT-PCR. Different lowercase letters indicate statistically significant differences among the groups within each panel (*p* < 0.05).

Enzymatic degradation analysis revealed that treatment with proteinase K, sodium periodate, and DNase I reduced biofilm biomass by 60%, 26%, and 13%, respectively (Figure 9A), indicating that extracellular proteins were the predominant component, followed by polysaccharides and eDNA. Moreover, different concentrations of MmEVs significantly suppressed the release of extracellular eDNA as well as the formation of extracellular polysaccharides and proteins compared with the control (Figure 9B–D, *p* < 0.05).

qRT-PCR analysis revealed that MmEVs significantly reduced the expression of key biofilm-associated genes in *S. aureus*. At MBIC and 1/2 MBICs, the expression of *sarA*, *icaB*, *fnbA*, *clfB*, and *cidA* was markedly reduced (all *p* < 0.05). At 1/4 MBIC, *sarA*, *icaB*, *cidA*, and *fnbA* remained significantly decreased, whereas *clfB* showed no significant change. Expression of the housekeeping gene *gyrB* was unaffected at all tested concentrations (Figure 9E).

### 3.10. Small RNA Sequencing and Target Prediction of mEV-Derived miRNAs

**Figure 10:**
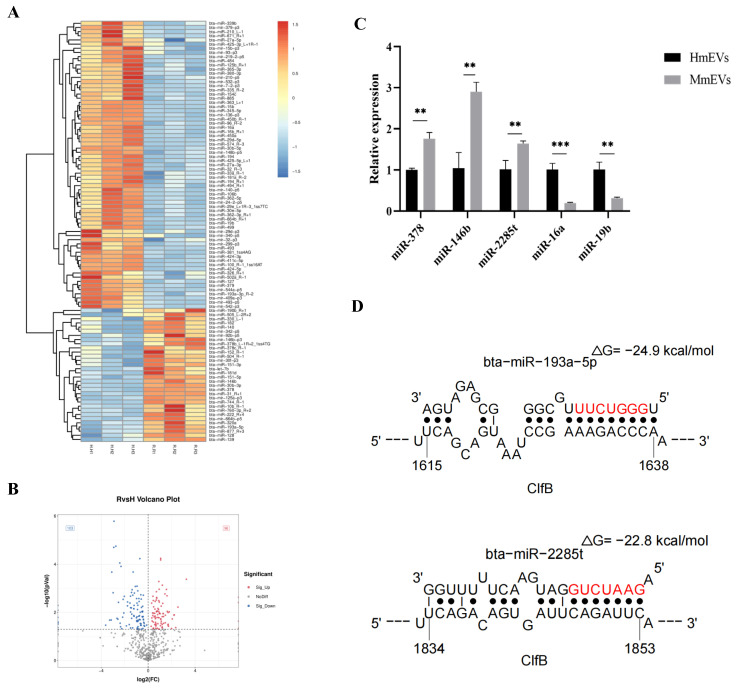
Sequencing and analysis of miRNAs in mEVs. (**A**) Heat map of the differentially expressed miRNAs between HmEVs and MmEVs. (**B**) Volcano map of the differentially expressed miRNAs between HmEVs and MmEVs. (**C**) Verification of the five differentially expressed miRNAs in mEVs by RT-qPCR (**: *p* < 0.01, ***: *p* < 0.001). (**D**) Schematic diagram of the putative binding sites of the seed sequence of miR-193a-5p and miR-2285t in *S. aureus*.

Since MmEVs consistently exhibited stronger inhibitory activity than HmEVs, we performed small RNA sequencing to identify potential molecular mediators. The sequencing analysis revealed that 96 miRNAs were upregulated and 103 were downregulated in MmEVs compared with HmEVs. The differentially expressed miRNAs were visualized using a heat map and volcano plot (Figure 10A,B). Five representative candidates (miR-378, miR-146b, miR-2285t, miR-16a, and miR-19b) were further validated by RT-qPCR, and the results were consistent with the sequencing data (Figure 10C). Bioinformatic analysis predicted that several of these miRNAs may target *S. aureus* virulence-related genes; notably, miR-193a-5p and miR-2285t were predicted to target *clfB* (Figure 10D), but they do not establish direct miRNA-mediated regulation. These findings provide a set of candidate mEV-derived miRNAs for subsequent functional studies on their roles in antibacterial and antibiofilm effects.

## 4. Discussion

Extracellular vesicles (EVs) have attracted increasing attention owing to their diverse biological functions. Previous studies have demonstrated that EVs derived from various sources can exert antimicrobial effects, including inhibition of bacterial growth and suppression of biofilm formation [23]. For example, camel milk exosomes suppress the growth of Gram-negative bacteria and fungi. However, the antimicrobial mechanisms of EVs remain insufficiently defined.

Reactive oxygen species (ROS) represent a double-edged sword in bacterial physiology: at low levels they serve as signaling molecules, whereas excessive accumulation leads to oxidative damage. To counteract oxidative stress, *S. aureus* relies on antioxidant enzymes such as superoxide dismutase (SOD), catalase (CAT), and peroxidase (POD) [24]. In this study, both HmEVs and MmEVs significantly increased ROS levels in *S. aureus* while simultaneously reducing the activities of SOD, CAT, and POD, thereby weakening its antioxidant defenses. Inhibition of ATPase activity further indicated impairment of energy metabolism and membrane function. Together, these results indicate that mEVs destabilize the antioxidant and metabolic balance of *S. aureus*, leading to reduced bacterial viability. Although our results show that mEVs exert strong inhibitory effects on *S. aureus* in vitro, under physiological conditions mEVs alone may not be sufficient to completely prevent infection. This apparent discrepancy likely reflects the complexity of the mammary gland environment, including dynamic changes in milk composition and flow, the multifactorial etiology and pathogen load of mastitis, and host immune and tissue factors that can outweigh or mask the modulatory effects of mEVs. Previous reports have shown that exosomes secreted by polymorphonuclear leukocytes (PMNs) infected with *S. aureus* exhibit pronounced antibacterial activity, whereas exosomes from uninfected cells show little or no such effect [21]. Consistent with this, we observed that MmEVs derived from mastitic cows displayed stronger inhibitory effects on *S. aureus* than HmEVs from healthy cows in multiple assays, including growth inhibition, ROS accumulation, and suppression of antioxidant enzyme activities, which may be related to stress-induced modulation of exosomal cargo, a phenomenon widely reported under pathological conditions [25]. These observations suggest that mastitis-associated remodeling of milk EV cargo could enhance the antimicrobial potential of MmEVs.

Biofilm formation is another key virulence strategy of *S. aureus*. Its extracellular matrix, mainly composed of polysaccharides, proteins, and extracellular DNA (eDNA), provides structural stability and protects bacteria from host defenses and antimicrobials [26]. The development of biofilms is tightly regulated by multiple genes, including *sarA*, *icaB*, *fnbA*, *clfB*, and *cidA* [27,28,29,30,31]. Our study showed that MmEV treatment significantly reduced extracellular polysaccharides, proteins, and eDNA within biofilms, accompanied by reduced expression of these biofilm-associated genes. Taken together, these observations are consistent with impaired biofilm development following MmEV exposure at both the level of extracellular matrix production and the expression of biofilm-associated genes, although the precise regulatory pathways underlying these changes remain to be clarified. Supporting evidence from other sources reinforces this notion, as *Streptococcus epidermidis* exosomes were shown to inhibit MRSA biofilm formation [32], bovine colostrum exosomes suppressed *S. aureus* biofilms [33], and bee-derived vesicles reduced biofilm formation in Gram-positive bacteria [14].

Importantly, our small RNA sequencing further revealed distinct miRNA profiles between HmEVs and MmEVs. Bioinformatic analysis predicted that several differentially expressed miRNAs, including miR-193a-5p and miR-2285t, may target *clfB*, a gene essential for bacterial adhesion and biofilm development. These associations, together with the phenotypic differences between HmEVs and MmEVs, are consistent with the hypothesis that mEV-derived miRNAs may contribute to antibacterial activity by modulating bacterial gene expression. However, we did not directly demonstrate uptake, stability, or target regulation of specific miRNAs in *S. aureus* in this study. Cross-kingdom regulation of gene expression by exosomal miRNAs has been increasingly recognized, with examples including dietary plant-derived miRNAs modulating mammalian gene expression and host-derived miRNAs influencing bacterial physiology [34,35]. In this context, miRNAs that are selectively enriched in MmEVs and predicted to target *S. aureus* genes involved in adhesion, biofilm formation, and stress adaptation can be regarded as candidate regulators that may contribute to the enhanced antibacterial phenotype of MmEVs, although their precise roles remain to be validated experimentally. Thus, vesicular miRNA signatures should be interpreted as correlating with changes in biofilm-associated gene expression rather than proving direct miRNA-mediated regulation, and they provide a set of candidate mEV-derived miRNAs for subsequent functional studies on their roles in antibacterial and antibiofilm effects. However, direct experimental evidence for the uptake of milk EV-derived miRNAs by bacteria and for their regulation of specific targets is still lacking.

Taken together, these findings indicate that mEVs act as multi-target modulators of *S. aureus* physiology, simultaneously perturbing oxidative stress defenses, energy metabolism, and biofilm formation. By directly comparing EVs from healthy and mastitic cows, our study provides evidence that mastitis is associated with a functional reshaping of the milk EV compartment, resulting in MmEVs with enhanced antibacterial activity against both a reference strain and clinical isolates. This extends previous observations that exosomes from activated immune cells or colostrum can inhibit *S. aureus* and other pathogens, by showing that disease-associated changes in milk EV cargo may represent an additional layer of host defense within the mammary gland. From a translational perspective, these results suggest that mEVs, or their key molecular cargos, could be further explored as templates or adjuvants for the development of residue-free strategies to limit *S. aureus* overgrowth and biofilm formation in the dairy industry. However, in this study we did not directly compare the antibacterial and antibiofilm effects of mEVs with conventional antimicrobials commonly used for bovine mastitis, nor did we quantify the natural concentration and composition of mEVs in mastitic milk; these aspects will require dedicated in vivo and comparative studies before clinical translation can be considered.

Despite these promising results, several limitations should be acknowledged. First, the doses of mEVs used in this study were selected to elicit measurable antibacterial and antibiofilm effects and to define in vitro dose–response relationships, and were not directly matched to the physiological levels of mEVs present in an equivalent volume of milk. Second, although the isolation protocol produced EV-enriched fractions confirmed by TEM, NanoFCM and exosomal marker expression, co-isolation of non-vesicular milk components cannot be fully excluded in the absence of density-gradient ultracentrifugation or quantitative purity metrics, and heat-inactivated or RNase-/proteinase-treated mEVs were not included as additional controls; consequently, the precise cargos responsible for the observed effects remain to be defined. Third, the proposed contribution of mEV-derived miRNAs to the observed antibacterial and antibiofilm phenotypes is inferred from differential expression profiles and in silico target prediction, rather than from direct evidence of miRNA transfer into *S. aureus* or modulation of bacterial gene expression. These miRNA-related observations should therefore be regarded as preliminary, hypothesis-generating leads that will need to be tested using miRNA mimics in combination with bacterial transcriptome or targeted gene-expression analyses. In addition, the virulence genotypes and detailed antimicrobial resistance profiles of the clinical *S. aureus* isolates were beyond the scope of this study, and future work integrating EV characterization with genomic and resistance profiling of clinical isolates will be important to strengthen the clinical relevance of our findings. Finally, in vivo studies will be essential to establish the therapeutic potential of mEVs in preventing or treating *S. aureus* infections.

## 5. Conclusions

In summary, this study shows that mEVs inhibit the growth and biofilm formation of Staphylococcus aureus in vitro. These effects were associated with induction of oxidative stress, suppression of extracellular matrix components, and reduced biofilm-associated gene expression. Marked differences in miRNA expression profiles between HmEVs and MmEVs, together with target prediction, suggest that several vesicular miRNAs may contribute to these antibacterial and antibiofilm phenotypes and thus represent candidates for future functional validation. These results provide additional insight into EV-mediated host–pathogen interactions in the bovine mammary gland. Our findings indicate that bovine mEVs have potential to serve as natural antimicrobial nanovesicles and residue-free adjuncts for mastitis control, although their clinical utility remains to be established in vivo.

## Data Availability

The original contributions presented in this study are included in the article. Further inquiries can be directed to the corresponding author(s).

## References

[1] Zaatout N., Ayachi A., Kecha M. (2020). *Staphylococcus aureus* persistence properties associated with bovine mastitis and alternative therapeutic modalities. J. Appl. Microbiol..

[2] Pérez V.K.C., Costa G.M.D., Guimarães A.S., Heinemann M.B., Lage A.P., Dorneles E.M.S. (2020). Relationship between virulence factors and antimicrobial resistance in *Staphylococcus aureus* from bovine mastitis. J. Glob. Antimicrob. Resist..

[3] Seethalakshmi P.S., Rajeev R., Kiran G.S., Selvin J. (2020). Promising treatment strategies to combat *Staphylococcus aureus* biofilm infections: An updated review. Biofouling.

[4] Tromp A.T., Van Strijp J.A.G. (2020). Studying Staphylococcal Leukocidins: A Challenging Endeavor. Front. Microbiol..

[5] Van Boeckel T.P., Brower C., Gilbert M., Grenfell B.T., Levin S.A., Robinson T.P., Teillant A., Laxminarayan R. (2015). Global trends in antimicrobial use in food animals. Proc. Natl. Acad. Sci. USA.

[6] Shao K., Yang Y., Gong X., Chen K., Liao Z., Ojha S.C. (2025). Staphylococcal Drug Resistance: Mechanisms, Therapies, and Nanoparticle Interventions. Infect. Drug Resist..

[7] Zhang Y., Liao T., Wang G., Xu J., Wang M., Ren F., Zhang H. (2023). An ultrasensitive NIR-IIa’ fluorescence-based multiplex immunochromatographic strip test platform for antibiotic residues detection in milk samples. J. Adv. Res..

[8] Jank L., Martins M.T., Arsand J.B., Motta T.M.C., Feijó T.C., Castilhos T.d.S., Hoff R.B., Barreto F., Pizzolato T.M. (2017). Liquid Chromatography–Tandem Mass Spectrometry Multiclass Method for 46 Antibiotics Residues in Milk and Meat: Development and Validation. Food Anal. Methods.

[9] Isaac R., Reis F.C.G., Ying W., Olefsky J.M. (2021). Exosomes as mediators of intercellular crosstalk in metabolism. Cell Metab..

[10] O’bRien K., Breyne K., Ughetto S., Laurent L.C., Breakefield X.O. (2020). RNA delivery by extracellular vesicles in mammalian cells and its applications. Nat. Rev. Mol. Cell Biol..

[11] Meldolesi J. (2018). Exosomes and Ectosomes in Intercellular Communication. Curr. Biol..

[12] Fang Y., Wang Z., Liu X., Tyler B.M. (2022). Biogenesis and Biological Functions of Extracellular Vesicles in Cellular and Organismal Communication With Microbes. Front. Microbiol..

[13] Sabatke B., Rossi I.V., Bonato L., Fucio S., Cortés A., Marcilla A., Ramirez M.I. (2025). Host–Pathogen Cellular Communication: The Role of Dynamin, Clathrin, and Macropinocytosis in the Uptake of Giardia-Derived Extracellular Vesicles. ACS Infect. Dis..

[14] Schuh C.M.A.P., Aguayo S., Zavala G., Khoury M. (2019). Exosome-like vesicles in Apis mellifera bee pollen, honey and royal jelly contribute to their antibacterial and pro-regenerative activity. J. Exp. Biol..

[15] Yu S., Zhao Z., Xu X., Li M., Li P. (2019). Characterization of three different types of extracellular vesicles and their impact on bacterial growth. Food Chem..

[16] Hiemstra T.F., Charles P.D., Gracia T., Hester S.S., Gatto L., Al-Lamki R., Floto R.A., Su Y., Skepper J.N., Lilley K.S. (2014). Human urinary exosomes as innate immune effectors. J. Am. Soc. Nephrol..

[17] He Y., He Z., Leone S., Liu S. (2021). Milk Exosomes Transfer Oligosaccharides into Macrophages to Modulate Immunity and Attenuate Adherent-Invasive, *E. coli* (AIEC) Infection. Nutrients.

[18] Ogunnaike M., Wang H., Zempleni J. (2021). Bovine mammary alveolar MAC-T cells afford a tool for studies of bovine milk exosomes in drug delivery. Int. J. Pharm..

[19] Yang H., Wuren T., Zhai B., Liu Y., Er D. (2024). Milk-derived exosomes in the regulation of nutritional and immune functions. Food Sci. Nutr..

[20] Yáñez-Mó M., Siljander P.R.-M., Andreu Z., Bedina Zavec A., Borràs F.E., Buzas E.I., Buzas K., Casal E., Cappello F., Carvalho J. (2015). Biological properties of extracellular vesicles and their physiological functions. J. Extracell. Vesicles.

[21] Timar C.I., Lőrincz M., Csepanyi-Komi R., Vályi-Nagy A., Nagy G., Buzás E., Iványi Z., Kittel Á., Powell D.W., McLeish K.R. (2013). Antibacterial effect of microvesicles released from human neutrophilic granulocytes. Blood.

[22] Luo Y., Bi J., Lin Y., He J., Wu S., Zhang Y., Wang Y., Song S., Guo H. (2023). Milk-derived small extracellular vesicles promote bifidobacteria growth by accelerating carbohydrate metabolism. LWT.

[23] Brakhage A.A., Zimmermann A.-K., Rivieccio F., Visser C., Blango M.G. (2021). Host-derived extracellular vesicles for antimicrobial defense. Microlife.

[24] Bastos M.L.C., Ferreira G.G., Kosmiscky I.O., Guedes I.M.L., Muniz J.A.P.C., Carneiro L.A., Peralta Í.L.C., Bahia M.N.M., Souza C.O., Dolabela M.F. (2025). What Do We Know About *Staphylococcus aureus* and Oxidative Stress? Resistance, Virulence, New Targets, and Therapeutic Alternatives. Toxics.

[25] Li X., Cao Y., Xu X., Wang C., Ni Q., Lv X., Yang C., Zhang Z., Qi X., Song G. (2023). Sleep Deprivation Promotes Endothelial Inflammation and Atherogenesis by Reducing Exosomal miR-182-5p. Arterioscler. Thromb. Vasc. Biol..

[26] Yan J., Bassler B.L. (2019). Surviving as a Community: Antibiotic Tolerance and Persistence in Bacterial Biofilms. Cell Host Microbe.

[27] Cramton S.E., Gerke C., Schnell N.F., Nichols W.W., Gotz F. (1999). The intercellular adhesion (ica) locus is present in *Staphylococcus aureus* and is required for biofilm formation. Infect. Immun..

[28] Tormo M.A., Martí M., Valle J., Manna A.C., Cheung A.L., Lasa I., Penadés J.R. (2005). SarA is an essential positive regulator of Staphylococcus epidermidis biofilm development. J. Bacteriol..

[29] Gries C.M., Biddle T., Bose J.L., Kielian T., Lo D.D. (2020). *Staphylococcus aureus* Fibronectin Binding Protein A Mediates Biofilm Development and Infection. Infect. Immun..

[30] Kashi M., Noei M., Chegini Z., Shariati A. (2024). Natural compounds in the fight against *Staphylococcus aureus* biofilms: A review of antibiofilm strategies. Front. Pharmacol..

[31] Rice K.C., Mann E.E., Endres J.L., Weiss E.C., Cassat J.E., Smeltzer M.S., Bayles K.W. (2007). The *cidA* murein hydrolase regulator contributes to DNA release and biofilm development in *Staphylococcus aureus*. Proc. Natl. Acad. Sci. USA.

[32] Chunxing X., Jingdi C., Xiang L., Ruicong W., Yu C., Yanwu L., Xiang H., Jingjing Z., Taoran W., Jiakai G. (2025). The inhibitory effect of extracellular vesicles derived from *S. epidermidis* on MRSA biofilms. Int. J. Pharm..

[33] Ahn G., Shin W.R., Lee S., Yoon H.W., Choi J.W., Kim Y.H., Ahn J.Y. (2023). Bovine Colostrum Exosomes Are a Promising Natural Bacteriostatic Agent against *Staphylococcus aureus*. ACS Infect. Dis..

[34] Zhang L., Hou D., Chen X., Li D., Zhu L., Zhang Y., Li J., Bian Z., Liang X., Cai X. (2012). Exogenous plant MIR168a specifically targets mammalian LDLRAP1: Evidence of cross-kingdom regulation by microRNA. Cell Res..

[35] Liu S., da Cunha A.P., Rezende R.M., Cialic R., Wei Z., Bry L., Comstock L.E., Gandhi R., Weiner H.L. (2016). The Host Shapes the Gut Microbiota via Fecal MicroRNA. Cell Host Microbe.

